# Predicting Cardiovascular Disease in Psychiatric Patients: Machine Learning with Electronic Health Records

**DOI:** 10.1192/j.eurpsy.2022.1744

**Published:** 2022-09-01

**Authors:** M. Bernstorff, A. Danielsen, S. Dinesen

**Affiliations:** Børglumvej 11, Department For Affective Disorders, Aarhus N, Denmark

## Abstract

**Introduction:**

Cardiovascular disease (CVD) causes staggering losses in quality adjusted life years worldwide.^1^ Among patients in the Danish psychiatric hospital setting, heart disease is associated with a decrease in life expectancy of 5.1 years.^2^ The causes underlying this association are likely manifold. For example, severe mental illness is associated with unhealthy lifestyle.^3^ Furthermore, psychiatrists may focus predominantly on the treatment of mental illness and have less emphasis on detection and prevention of physical illness.^4^ If patients at elevated risk of CVD are pointed out automatically, this may lead to better preventive medicine.

**Objectives:**

To predict which patients develop cardiovascular disease using machine learning.

**Methods:**

We obtained data on all psychiatric hospital contacts in the Central Denmark Region since the initiation of the current EHR system (MidtEPJ). These span from 2011 to 2021 and cover 120,000 patients, of which 3,000 patients developed severe CVD (stroke or coronary event) follow-up. We will train a variety of models (random forests, SVM, deep neural nets) to predict CVD within one year from a planned contact to hospital.

**Results:**

The modelling is currently underway, intermediary results are expected in January.

**Conclusions:**

We explore whether predicting CVD is feasible using state-of-the-art technologies and a uniquely detailed dataset. This may pave the way for machine learning to act as a clinical support decision system, since we’re only training on data that is available in a live, clinical context.

**References**

1: Khan 2019

2: Erlangsen 2017

3: Scott 2011

4: Fagiolini 2009

**Disclosure:**

No significant relationships.

